# Performance of general health workers in leprosy control activities at public health facilities in Amhara and Oromia States, Ethiopia

**DOI:** 10.1186/s12913-016-1329-2

**Published:** 2016-04-07

**Authors:** Tadiye Abeje, Edessa Negera, Eshetu Kebede, Tsegaye Hailu, Ismaile Hassen, Tsehainesh Lema, Lawrence Yamuah, Birru Shiguti, Melkamu Fenta, Megersa Negasa, Demissew Beyene, Kidist Bobosha, Abraham Aseffa

**Affiliations:** Armauer Hansen Research Institute, P.O.Box 1005, Ayer Tena Road, Addis Ababa, Ethiopia; ALERT Training Division, P.O.Box. 1005, Ayer Tena Road, Addis Ababa, Ethiopia; Federal Ministry of Health, P.O.Box 1234, Addis Ababa, Ethiopia; Oromia Regional Health Bureau, P.O.Box 24341, Addis Ababa, Ethiopia ᅟ; Amhara Regional Health Bureau, P.O.Box 495, Bahir Dar, Ethiopia ᅟ

**Keywords:** Ethiopia, General health workers, Knowledge, Attitude and practice, Leprosy, Leprosy management, Multidrug treatment

## Abstract

**Background:**

Leprosy is a chronic infectious disease of public health importance and one of the leading causes of permanent physical disability. Nevertheless, the drop in prevalence following multidrug therapy has resulted in the neglect of leprosy. The annual incidence of leprosy has remained the same in Ethiopia since decades with more than 76 % of the reported new cases coming from Oromia and Amhara Regional States. This study was aimed to assess the knowledge, attitude and skill of general health workers in leprosy control activities at public health facilities in Oromia and Amhara Regional States.

**Methods:**

A cross-sectional study was conducted from September 2011 to February 2012 at different public health facilities in selected eight zones in Oromia and Amhara Regional States. A multistage sampling method was used to obtain representative samples. High and low endemic zones for leprosy were included in the study in both regional states. Data were collected from general health workers through a structured self-administered questionnaire and at on-site assessment of their performance. Baseline socio-demographic data, health workers’ attitude towards leprosy and their knowledge and skill in the management of leprosy were assessed. Bloom’s cut off point was used to describe the knowledge and practical skills of the respondents while Likert’s scale was used for attitude assessment.

**Result:**

A total of 601 general health workers responsible for leprosy control activities at public health facilities were included in knowledge and attitude assessment and 83 of them were subjected to practical evaluation, with on-site observation of how they handle leprosy patients. These included medical doctors (4 %), health officers and nurses with Bachelor degree in Science (27 %), clinical nurses with diploma (66 %) and health assistants (2.8 %). The median age of the respondents was 26.0 years and females made up of 45 %. Generally the knowledge and skills of the respondents were found to be poor while attitude towards leprosy was positive for the majority of the respondents. The result showed that 519 (86.3 %) had poor knowledge. Overall 155 (25.8 %) of the respondents had positive attitude towards leprosy while 205 (34.1 %) had intermediate (mixed) attitude and 241 (40.1 %) had negative attitude to the disease. Among 83 respondents assessed for diagnosis of leprosy only 15(18.0 %) diagnosed leprosy correctly. Variation in knowledge and attitude indicated a significant difference (*p* < 0.05) among different health institutions, professions, gender, in-service training and years of experience.

**Conclusion:**

The current finding underlines that although leprosy control activities are integrated to the general health services in the country, the knowledge and skills of leprosy diagnosis, treatment and management by health workers was unsatisfactory. Hence, attention should be given to develop training strategies that can improve health worker knowledge and promote better leprosy management at public health facilities. This could be achieved through pre-service and in-service training and giving adequate emphasis to leprosy related practical work and continuous follow- up.

## Background

In Ethiopia, the prevalence of leprosy has dropped from 80,927 cases in 1982 to 2,944 cases in 2012 while the incidence rates dropped from 6,243 to only 3,776 in the same period [1]. Although, according to the World Health Organization (WHO), Ethiopia reached the leprosy elimination target of 1 case/10,000 population in 1999, the incidence has not changed appreciably [2]. In addition, there are pocket areas that report significantly high number of cases annually. For instance, the Kokosa district health bureau (West Arsi Zone) has reported 62 new leprosy cases in 2011/2012. More than 76 % of the national leprosy cases were notified from Oromia and Amhara regional states in 2012 [3].

In Ethiopian health care system, leprosy was treated vertically by leprosy specialized personnel until 2001 at leprosy specialized hospitals. The leprosy control program has been fully integrated into the general health services by the end of 2001 to ensure that patients are diagnosed at early stage and complete the multidrug treatment (MDT) without accompanying disability [4]. This means that leprosy prevention and control activities became the responsibility of the general health service. However, this integration made that patients are seen by general health workers during outpatient visits rather than by leprosy specialized personnel in leprosy dedicated clinics which could lead to misdiagnosis and treatment [4].

The Ethiopian national health system is organized into a three-tier health service system which comprises a primary health care unit (a network of a health centre and five health posts), primary hospitals and specialized referral hospitals. Health posts deliver preventive and promotion services at house-hold levels, accesses to immunization, family planning service, antenatal care and community based services such as HIV, TB, Malaria and other diseases [5].

Health posts refer leprosy suspects to health centres. Health centres diagnose and treat leprosy patients and they serve as the main health facility for initiating MDT and follow- up. They are also supposed to treat and manage mild reactions and refer severe reactions and complications. However, steroids for treatment of reactions are only available at hospitals and the treatment of mild reactions at health centres remains as unmet goal. Leprosy referral hospitals provide referral services for diagnosis and treatment and provide in-patient services. These leprosy referral hospitals are quite few and deal only with complicated cases such as leprosy reactions. Drugs are supplied quarterly to health facilities based on the previous quarter registered number of patients. Regular assessment is done at the woreda, zonal, regional and national levels where epidemiological and operational indicators for monitoring of the leprosy are calculated and compiled. Quarterly reports are completed according to the Ethiopian fiscal year. Tuberculosis (TB), Leprosy and TB/HIV collaborative activities eventually integrated into the Health Management Information System.

The emphasis in leprosy control will remain on providing diagnostic and treatment services that are equitably distributed, affordable, accessible, acceptable and good standard of quality. However, diagnostic and treatment service can be challenged by the health workers’ knowledge and skills in the recognition of the signs and symptoms of leprosy at an early stage of the disease [5–9]. Ignorance, lack of awareness, stigmatization, and poor interaction with the health service in the general population also prevent people from going to health facilities for taking health seeking action and treatment which leads to late diagnosis and nerve impairment among most leprosy patients [10]. The priority of national leprosy control program is to prevent the primary nerve damage which leads to disability. This can be achieved by early diagnosis and timely initiation of the correct treatment of leprosy by health workers [11–14].

Information on the performance of the general health workers engaged in leprosy control activities at public health facilities is important to increase the awareness of policy makers and stakeholders on how leprosy control activities are operating in the public health care system. It also measures the quality of health workers’ performance.

The knowledge, attitude and skills of general health workers in leprosy control activities has never been addressed since the integration of leprosy control program into the general health service in Ethiopia. Therefore, this study was designed to address the level of knowledge, attitude and skills of general health workers engaged in leprosy control activities at different levels of health facilities and provide information to policy makers and stakeholders.

## Methods

A facility-based cross-sectional study was conducted from September 2011 to February 2012 in the two largest regional states of Ethiopia (Oromia and Amhara Regional Sates) which contributed to 76 % of leprosy notifications in 2012 [3]. A multistage sampling method was used to get representative samples. Each region was divided into high and low endemic zones based on the mean of the last 3-years annual report of new leprosy cases detection and disability rates [3].

Two high endemic and two low endemic zones from each regions were selected by simple random sampling method. Jimma and East Hararge from Oromia region and South Wello and West Gojam from Amhara region were selected as high leprosy endemic zones while Borena and North Shewa from Oromia region and Waghimra and Kemissie from Amhara region were selected as low leprosy endemic zones. From each zone, 50 % of the districts (woreda) were randomly selected and from the selected districts, Fifty percent of the randomly selected health centres; all referral and district hospitals in each zone were included to the study. However, leprosy referral hospitals were excluded from the study.

Clinical nurses, health officers and medical doctors working at the selected public health facilities both in- and out-patient departments and one at the tuberculosis and leprosy (TBL) clinics were enrolled to the study. A carefully structured knowledge and attitude questionnaire were self-administered to individuals who were at the facility on the date of visit, working in-out patient department and TBL clinic. Assessments of the skills were made using a structured checklist for observation of the skills of one general health worker at TBL clinic by leprosy experts. The leprosy expert group consists of public experienced health officers, medical doctors and nurses in leprosy diagnosis and treatment. The skills of each selected health workers were assessed while he/she diagnoses the leprosy suspect or patient. But if there was no leprosy suspect at the time of visit, one of the data collectors simulated as leprosy suspect and was evaluated by the health worker. We used Bloom’s cut off point to measure knowledge and practice of the respondents and Likert’s scale to measure the attitude of the respondents [7]. Percent of correct response to a set of 15 knowledge questions was used for grading as follow as: 59 % or below (8/15), 60-80 % (9-12/15) and above 80 % (>12/15) were considered for low, medium and high knowledge of leprosy respectively. Similarly, out of 10 skill tests those who performed correctly in 5 or below 59 % (5/10), 60-80 % (5-8/10) and above 80 % (>8/10) were marked as unsatisfactory, satisfactory and excellent skill to diagnose leprosy respectively. For the attitude assessment, out of 10 attitude questions those who answered positive less than 39 % (3/10), 40-60 % (4-6/10) and above 60 % (>7/10) were considered as having a negative, intermediate and positive attitude towards leprosy respectively.

### Ethical considerations

The protocol was approved by the AHRI/ALERT Ethical Review Committee with the approval protocol number of P014/10. Support letters were obtained from the Amhara and Oromia regional health bureaus. The information sheet was read to the participants in groups and verbal consent was obtained from each of the participants. All personal information was kept confidential and reporting was made anonymous.

## Results

### Socio-demographic characteristics

A total of 601 general health workers responsible for leprosy control activities at public health facilities were included in the knowledge and attitude assessment and 83 of them were subjected to practical evaluation, with on-site observation while they were assessing patients for leprosy. The median age of the respondents was 26.0 years. The proportions of doctors, BSc health officers and nurses, diploma clinical nurses and health assistants were 4 %, 27.1 %, 66.1 % and 2.8 % respectively. The proportion of females was 45.4 % making the female to male ratio 0.8:1. Five-hundred six (84.0 %) of the respondents were working at health centres, 68 (11.0 %) at a district hospital and 27 (5.0 %) at zonal/referral hospitals. The median work experience of the respondents was 3.0 years (range: 0-42 years). Only 195 (32.4 %) of the respondents had received in-service training on leprosy (Table 1).

**Table 1:** Socio-demographic characteristics of the general health workers in leprosy control activities by type of institution, gender, profession and years of service in Oromia and Amhara Regional Sates, Ethiopia, February 2012

Variable	Characteristics	No	%
Sex	Male	328	54.6
	Female	273	45.4
Health profession	MD	24	4.0
	BSc (HO and Nurse)	163	27.1
	Diploma clinical nurse	397	66.1
	Health assistant	17	2.8
Median age by sex	Male	328	27.0
	Female	271	24.0
Year of service (experience)	0-9	451	75.0
	10-19	71	11.8
	20-29	35	5.8
	>30	44	7.3
In-service training in leprosy	Yes	195	32.4
	No	406	67.6
Respondents by institution	Health centre	506	84.2
	District Hospital	68	11.3
	referral Hospital	27	4.5
Median age by profession (Years)	MD	31	years
	BSc (HO and Nurse)	26	“
	Diploma clinical nurse	25	“
	Health assistant	33	“

### Knowledge of the respondents

The knowledge score of the respondents (health workers) was low for 519 (86.36 %), medium for 78 (13.0 %) and high for 4 (0.7 %). The level of knowledge was low for diploma clinical nurses and health assistants while doctors and BSc holders (Health officers and Nurses) relatively had either medium or low knowledge. The level of knowledge was better among respondents working at district and zonal hospitals than those working at health centres and the difference was statistically significant (*P* < 0.05). In-service trained health workers had a higher level of knowledge (*P* < 0.05) compared to those who did not receive in-service training (Table 2).

**Table 2:** The level of the knowledge of the general health workers in leprosy control activities by type of institution, gender, profession and years of service in Oromia and Amhara Regional Sates, Ethiopia, February 2012

Variable	Low	Medium	High	Total		
	N (%)	N (%)	N (%)	N	X^2^	P value
Health profession						
MD	15(62.5)	9(37.50)	0(0)	24	39.42	0.000
BSc(HO and Nurse)	124(76.1)	36(22.1)	3(1.8)	163		
Diploma clinical nurse	365(92.0)	31(7.8)	1(0.3)	397		
Health assistant	15(88.2)	2(11.8)	0(0)	17		
Type of Health institution						
Health centre	448(88.5)	54(10.7)	4(0.8)	506	16.2733	0.003
District hospital	52(76.5)	16(23.5)	0	68		
Zonal/referral hospital	19(70.4)	8(29.6)	0	27		
Gender (sex)						
Male	273(83.2)	52(15.9)	3(0.9)	328	6.5996	0.159
Female	246(90.1)	26(9.5)	1(0.4)	273		
Year of experience						
0-9	395(87.6)	54(12.0)	2(0.4)	451	7.5866	0.27
10-19	58(81.7)	11(15.5)	2(2.8)	71		
20-29	29(82.9)	6(17.1)	0	35		
>30	37(84.1)	7(15.9)	0	44		
Training in leprosy						
Yes	156(80.0)	35(18.0)	4(2.0)	195	8.0952	0.017
No	363(89.4)	43(10.6)	0(0.0)	406		
overall	519(86.4)	78(13.0)	4(0.7)	601		

The majority of health workers knew that leprosy is caused by bacteria, but 63 % of them thought it could be transmitted through casual contact and 79 % could not describe the main body parts affected by leprosy (skin and peripheral nerves). Seventy four percent of the health workers were not able to list down the cardinal signs of leprosy and 62 % could not specify the correct duration of treatment of leprosy. About 71 % the respondents did not know the pathophysiology of disability in leprosy. Only 17 % of them were able to correctly list the signs and symptoms of leprosy reaction but the overwhelming majority, 97 % did not know how to manage leprosy reactions.

### Attitude scores

Attitude was scored as positive in 155(25.8 %), intermediate in 205 (34.1 %) and negative in 241 (40.1 %). Attitude score was significantly associated with health institution, professional qualification, gender and previous in-service training (Table 3). About 58 % of the health workers expressed fear of contracting the disease while treating a leprosy patient who has deformity and/ulcer, even if they knew that the patient was on MDT. Seventy one percent of them preferred to use gloves when examining a leprosy suspect (a practice they did not follow for non-leprosy patients) and 21 % of them stated that they would not examine a case or a leprosy suspect at all for fear of contracting the disease. About 45 % health workers preferred to isolate leprosy cases from others.

**Table 3:** Attitude of the general health workers in leprosy control activities by type of institution, gender, profession and years of service in Oromia and Amhara Regional Sates, Ethiopia, February 2012

Variable	Negative	Intermediate	Positive	Total		
	N (%)	N (%)	N (%)	N	X^2^	P value
Health profession						
MD	2(8.3)	12(50.5)	10(41.7)	24	44.0292	0.000
BSc(HO and Nurses)	60(36.8)	63(38.7)	40(24.5)	163		
Diploma clinical nurse	170(42.8)	123(31.0)	104(26.2)	397		
Health assistant	9(52.9)	7(41.2)	1(5.9)	17		
Respondents by Type of health facility						
Health centre	205(40.5)	190(37.5)	111(21.9)	506		
District hospital	24(35.3)	12(17.6)	32(47.1)	68	9.1421	0.048
Zonal/referral hospital	12(44.4)	3(11.1)	12(44.4)	27		
Gender (sex)						
Male	116(35.4)	109(33.2)	103(31.4)	328	15.3424	0.004
Female	125(45.8)	96(35.2)	52(19.0)	273		
Year of experience						
0-9	184(40.8)	154(34.1)	113(25.1)	451	19.76	0.013
10-19	23(32.39)	30 (42.25)	18(25.35)	71		
20-29	13(37.1)	9(25.7)	13(37.1)	35		
>30	21(47.7)	12(27.3)	11(25.0)	44		
Training in leprosy						
Yes	62(31.8)	82(42.1)	51(26.1)	195	9.0006	0.011
No	179(44.1)	123(30.3)	104(25.6)	406		
Overall	241(40.1)	205(34.1)	155(25.8)	601		

### Skills for leprosy diagnosis

Among 83 respondents assessed for skills in the diagnosis of leprosy, only 15 (18.07 %) diagnosed leprosy correctly. The level of skills of the health workers varied according to health profession, year of experience and type of health institution (*P* < 0.05). However, variation was not significant by gender or previous in-service training (Table 4). On examining a skin of the suspect, 84 % of the health workers were unable to correctly perform sensation testing on the patch and sensory testing on the palm and soles. Health workers who could correctly perform voluntary muscle testing on the eyes, hands and feet were only 10 %, 9 % and 14 %, respectively. Eighty two percent of the health workers were unable to correctly classify leprosy cases during the actual clinical examination of leprosy suspects. Ninety one percent of them were unable to correctly grade the disability status of the patient and the majority of them (82 %) could not prescribe the correct treatment regimen.

**Table 4:** The level of the skills of the general health workers in leprosy diagnosis and treatment by type of institution, gender, profession and years of service in Oromia and Amhara Regional Sates, Ethiopia, February 2012

Variable	Unsatisfactory	Satisfactory	Excellent	Total		
	N (%)	N (%)	N (%)	N	X^2^	P value
Health profession						
MD	0	0	1(100)	1	19.9396	0.001
BSc(HO and Nurse)	7(53.8)	2(15.4)	4(30.8)	13		
Diploma clinical nurse	59(90.8)	4(6.2)	2(3.1)	65		
Health assistant	2(40.0)	0 (0)	3(60.0)	5		
Respondents by Type of health institution						
Health centre	64(87.7)	3(4.1)	6(8.2)	73	10.7185	0.030
District hospital	4(50.0)	2(25.0)	2(25.0)	8		
Zonal/referral hospital	0(0)	1(50.0)	1(50.0)	2		
Gender (sex)						
Male	32(80.0)	3(7.5)	5(12.5)	40	5.7196	0.221
Female	36(83.7)	3(7.0)	4(9.3)	43		
Year of experience						
0-9	52(88.1)	4(6.8)	3(5.1)	59	27.8339	0.000
10-19	12(100.0)	0(0)	0(0)	12		
20-29	1(100.0)	0(0)	0(0)	1		
>30	3(27.27)	2(18.18)	6(54.55)	11		
Training in leprosy						
Yes	45(84.9)	3(5.7)	5(9.4)	53	4.0795	0.130
No	23(76.7)	3(10.0)	4(13.3)	30		
Overall	68(81.9)	6(7.2)	9(10.8)	83		

## Discussion

Although leprosy control activities are integrated into the general health services in Ethiopia, the knowledge and skills of leprosy diagnosis, treatment and management of leprosy by general health workers were found to be unsatisfactory. This study showed that the majority of health workers had poor knowledge in recognition of the early signs and symptoms of leprosy, leprosy reaction and its management. The majority of them were unable to perform sensation and voluntary muscle testing. This may contribute to the prevalence of the relatively high proportion (14 %) of grade 2 disability in Ethiopia. The attitude of a significant proportion of health workers was also found to be unfavourable towards leprosy suspects and people affected by leprosy. This might discourage leprosy patients to self-report at public health facilities. They would prefer to go to holy baths and traditional healers or stay at home while their skin and nerve lesions worsen.

 Our findings have shown  that the performance of the health workers is associated with the level of qualification, in-service trainings and previous exposure to leprosy work. Almost 86 % of the health workers had low-level knowledge which could be attributed to little attention given during formal training, lack of practice after training and low number of leprosy cases for practice per site which needs further confirmation. This implies that increasing level of awareness about leprosy through training of health workers working at primary health care level and incorporating meaning full leprosy education into the curricula of medical and paramedical courses are some of the tasks to be accomplished by the stakeholders [4].

The absence of high level of knowledge among general health workers working at district and zonal hospitals is probably due to the fact that most general health workers at these health facilities focused on other diseases and hence are not motivated to update themselves in leprosy. The relatively high percentage of positive attitude among general health workers working at health centres provides a good opportunity to improve through emphasis on in-service training since majority of leprosy cases are seen and treated at health centres.

As the years of service increased, it appeared that there is an increasing trend towards a lower level of knowledge and an increased negative attitude to leprosy care. Therefore, involving these health workers in leprosy control activities necessitates in-service training and additional refreshment courses to increase their awareness and improve their attitude.

## Conclusion

This study showed that the majority of health workers had poor knowledge in recognition of the early signs and symptoms of leprosy, reaction and its management. The attitude of health workers was also found to be unfavourable towards leprosy suspects and people affected by leprosy. The skills of general health workers was also found to unsatisfactory; the majority of them were unable to perform sensation and voluntary muscle testing. The study also showed that the association of the performance of health workers with the level of qualification, in-service trainings and previous exposure to leprosy diagnosis and treatment. In order to improve the skill, knowledge and attitude of the health workers, continuous training and health education on leprosy should be emphasized at pre-service and in-service levels.

## Abbreviations

AHRI: Armauer Hansen Research Institute
BSc: Bachelor science
HO: Health officer
MD: Medical doctor
MDT: Multidrug treatment
TB: Tuberculosis
TBL: Tuberculosis and leprosy
WHO: World Health Organization
